# Molecular pharmacology of the onco-TRP channel TRPV6

**DOI:** 10.1080/19336950.2023.2266669

**Published:** 2023-10-15

**Authors:** Arthur Neuberger, Alexander I. Sobolevsky

**Affiliations:** Department of Biochemistry and Molecular Biophysics, Columbia University, New York, NY, USA

**Keywords:** TRPV6, TRP channels, cancer, inhibitor, cryo-EM, structural biology

## Abstract

TRPV6, a representative of the vanilloid subfamily of TRP channels, serves as the principal calcium uptake channel in the gut. Dysregulation of TRPV6 results in disturbed calcium homeostasis leading to a variety of human diseases, including many forms of cancer. Inhibitors of this oncochannel are therefore particularly needed. In this review, we provide an overview of recent advances in structural pharmacology that uncovered the molecular mechanisms of TRPV6 inhibition by a variety of small molecules, including synthetic and natural, plant-derived compounds as well as some prospective and clinically approved drugs.

## Introduction

### Physiology and pathophysiology of TRPV6

Transient receptor potential (TRP) channels are versatile membrane proteins that can be divided into master regulators (gatekeepers) of ion homeostasis and sensory transducers that respond to changes in temperature (including noxious and innocuous heat and cold), pH, mechanical stress, and irritant chemicals, like pungent compounds of hot chili peppers (capsaicin), mint (menthol), Japanese horseradish wasabi (isothiocyanate), oregano and thyme (carvacrol), or garlic and onions (allicin). Whilst the former help us to regulate quintessential cell functions, the latter play an important role in our interaction with the environment and contribute to our ability to make healthy choices.

When a TRP channel is activated, it undergoes conformational changes, transitioning into a state, in which its transmembrane pore opens for conductance of ions that pass between extracellular and intracellular spaces. Some channels open their pores only in response to an agonist or a physical stimulus. Other channels, like TRPV6, are constitutively active, meaning that they always “flicker” between conducting and non-conducting conformations, with the probability of conducting conformation (channel open probability) dependent on the presence of different regulating factors in the environment. The development of efficient protocols for expression and purification of TRP channels for cryo-electron microscopy [1] in combination with methods of functional analysis, such as single-channel and whole-cell current recordings and fluorescence-based calcium imaging, mutagenesis and molecular dynamics (MD) simulations have proven to be effective tools to uncover the molecular mechanisms of TRP channel regulation by different environmental factors as well as natural and synthetic compounds. The ability to study the mechanisms of activation and inhibition at the molecular level is especially important given that aberrancies in the TRP channel function are often associated with various human diseases.

TRPV6, previously referred to as ECaC2 or CaT1, is a representative of the vanilloid subfamily of TRP channels – one of the seven TRP channel subfamilies. TRPV6 is highly selective to Ca^2+^ (P_Ca_/P_Na_ >100) [2–5] and its constitutive activity is regulated positively by membrane lipids, such as phosphatidylinositol 4,5-bisphosphate (PIP_2_) [6–8], and negatively by Mg^2+^, which is at least partially responsible for the strong inward current rectification displayed by these channels [9,10], as well as calmodulin (CaM), which causes Ca^2+^-dependent inactivation [11–13] via very specific protein–protein interaction [14]. TRPV6 is the principal calcium uptake channel in the gut, capturing diet-delivered calcium ions [5,15–17]. Being highly concentrated at the microvilli tips of the lumen-facing epithelial cells of the duodenum, TRPV6 also expresses in proximal jejunum, cecum and colon, as well as blood, bone, epididymis, esophagus, kidney, liver, lung, pancreas, pituitary, placenta, prostate, salivary gland, stomach, sweet gland, teeth, testis, tongue, trachea, and uterus cells [18–24].

As TRPV6 represents an essential gatekeeper and master regulator of calcium uptake, its dysregulation results in disturbed calcium homeostasis. It has been found that knockout *Trpv6*^–/–^ mice show defective absorption of Ca^2+^ in the intestine, an increase in urinary Ca^2+^ excretion, a decrease in femoral bone mineral density, lower body weight, alopecia, dermatitis, and severely impaired male fertility [25–29]. Numerous mutations in the human TRPV6 gene have been linked to transient neonatal hyperparathyroidism, skeletal under-mineralization and dysplasia, hypercalciuria, chronic pancreatitis, various reproductive diseases, Pendred syndrome and Crohn’s-like disease [30–42].

Calcium uptake and signaling play a central role in cancer development and proliferation [43]. Not surprisingly, therefore, TRPV6 was found overexpressed in some of the most aggressive human cancer types, including breast, prostate, colon, ovarian, thyroid, endometrial cancers, and leukemia [23,43–49]. For instance, Peleg *et al*. found that overexpression of TRPV6 results in colonic crypt hyperplasia in mice and colon cancer cell proliferation in humans [47]. Accordingly, suppression of TRPV6 activity was proposed to underlie cancer protective effects in the colon when following a calcium-rich diet [47]. Moreover, 93% of biopsies uncovered higher TRPV6 levels in invasive compared to noninvasive tumor areas [50]. Furthermore, an ancestral variant of this oncochannel has emerged as a driver of higher incidence, higher mortality, and more aggressive forms of cancer in people of African descent [51].

A search for small-molecule inhibitors and ion channel blockers of TRPV6 as potential drugs identified a number of lead compounds, most of which are either not potent or selective enough to be used as medicines [52–58]. The most promising examples are a representative of TH-1177 compounds, which extends the average life span of mice carrying prostate tumors [53], and one of the (4-phenylcyclohexyl)piperazine derivatives (PCHPDs) that blocks TRPV6 transport function in cells as assessed by the reduction of Cd^2+^ toxicity but shows reduced off-target effects, in particular, suppressed *h*ERG inhibition [57]. Nevertheless, none of the small-molecule TRPV6 inhibitors has yet made it through clinical trials and became an approved drug. In this regard, the most advanced is a 13-amino acid peptide SOR-C13 derived from Soricidin, a 54-residue peptide found in the paralytic venom of the northern short-tailed shrew *Blarina brevicauda*, that has completed Phase I clinical safety trial [59–61]. SOR-C13 was reported to inhibit ovarian and prostate cancer growth *in vitro* and *in vivo* and demonstrated a potential for in vivo diagnostic cancer imaging. While SOR-C13 is believed to directly interact with TRPV6, no supporting structural information is available. Drugs capable of regulating TRPV6 are therefore urgently needed.

This review highlights recent advances in molecular pharmacology of TRPV6 achieved by combining cryo-electron microscopy with other biophysical and biochemical approaches.

### TRPV6 structural architecture

**Figure 1.**
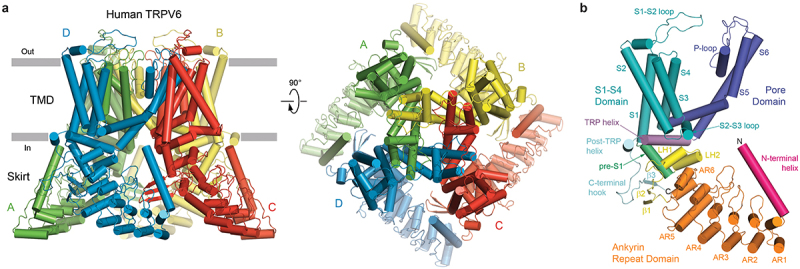
**TRPV6 architecture and domain organization.** a, side (left) and top (right) views of human TRPV6 tetramer (PDB ID: 7S88), with subunits (A-D) shown in different colors (green, yellow, red, and blue). b, a single TRPV6 subunit, with domains shown in different colors and labeled. Adapted from [62].

Numerous apo-state structures of human and rat TRPV6 have been determined using both cryo-electron microscopy and X-ray crystallography [62–66]. TRPV6 is assembled of four subunits and contains a transmembrane domain (TMD) forming the central ion channel pore and an intracellular “skirt” built of ankyrin repeat domains, which are connected by three-stranded β-sheets, N-terminal helices, and C-terminal hooks [62] (Figure 1). The amphipathic TRP helices, which represent a characteristic feature of the TRP channel family and connect the TMD to the C-terminal hook, run nearly parallel to the membrane inner leaflet and interact with both the TMD and the skirt. The TMD is composed of six transmembrane helices (S1–S6) and a pore loop (P-loop) between S5 and S6. A bundle of the first four transmembrane helices represents the S1–S4 domain. A domain homologous to S1-S4 in voltage-gated ion channels acts as a voltage sensor [67]. The pore-forming domain of each protomer includes S5, P-loop and S6, and leans against the S1–S4 domain of the neighboring subunit in a domain-swapped arrangement [63,68].

The outer TRPV6 pore entry starts with an extracellular vestibule (Figure 1). Four symmetry-related extracellular vestibule recruitment sites [69] were previously identified there based on anomalous difference Fourier peaks for Ba^2+^ and Gd^3+^ ions [62,68]. These sites are contributed by negatively charged residues aspartates and glutamates. Although no strong anomalous difference Fourier peaks at the recruitment sites were found for Ca^2+^, the highly electronegative surface of the extracellular vestibule is almost certainly involved in the recruitment of all types of cations to the outer pore entrance [62]. In agreement with the isothermal titration calorimetry for Gd^3+^, cation affinity to the recruitment sites is lower than to the binding sites in the pore [62]. At the extracellular pore entry site [69], strong anomalous peaks coordinated by side chains of the highly conserved aspartates (D542 in hTRPV6 and D541 in rTRPV6) were identified for the permeant cations Ca^2+^ and Ba^2+^, and for the channel blocker Gd^3+^ ion [62,68].

## Lipid activators of TRPV6

**Figure 2.**
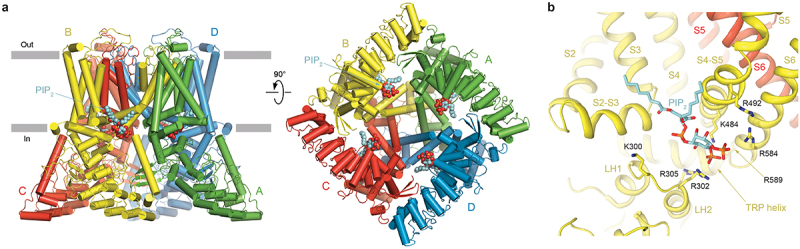
**Activation of TRPV6 by PIP**_**2**_. a, side (left) and bottom (right) views of the open, apo-state hTRPV6 (PDB ID: 7S88), with subunits (A-D) colored green, yellow, red, and blue, and PIP_2_ molecules (space-filling models) projected from the PIP_2_-bound structure of TRPV5 (PDB ID: 6DMU). b, expanded view of the PIP_2_ binding site, with the PIP_2_ molecule and residues surrounding the head group of PIP_2_ shown as stick models. Adapted from [71,72].

PIP_2_ was shown to act as a TRPV6 activator that is required to maintain the constitutive activity of this channel [6]. While TRPV6 structures in complex with PIP_2_ are not yet available, computational modeling and mutational analysis suggested that PIP_2_ binds to TRPV6 at the same site as in TRPV5, where it also serves as an activator [70,71]. In TRPV5, PIP_2_ binds to the S2-S3 site [69] contributed by the linker region (linker helices LH1 and LH2), S2-S3 and S4-S5 linkers and S6 helix [71]. Due to structural similarity of TRPV5 and TRPV6 in this region, it is easy to project PIP_2_ binding to TRPV6 (Figure 2). Like in TRPV5, the positively charged residues K300, R302, R305, K484, R492, R584 and R589 are expected to either directly contribute to S2-S3 site or pH-dependently interact with PIP_2_ when this lipid approaching its binding location [71,73,74]. Interestingly, long-chain acyl-coenzyme A (LC-CoA), a crucial metabolic intermediate that plays important cellular regulatory roles, can also activate TRPV5 and TRPV6 channels [75]. Based on the cryo-EM structure of the TRPM5-LC-CoA complex, LC-CoA binds to the same S2-S3 site and PIP_2_ can no further activate TRPV5 or TRPV6 in the presence of LC-CoA [75].

## Endogenous inhibitors of TRPV6

### Calmodulin

**Figure 3.**
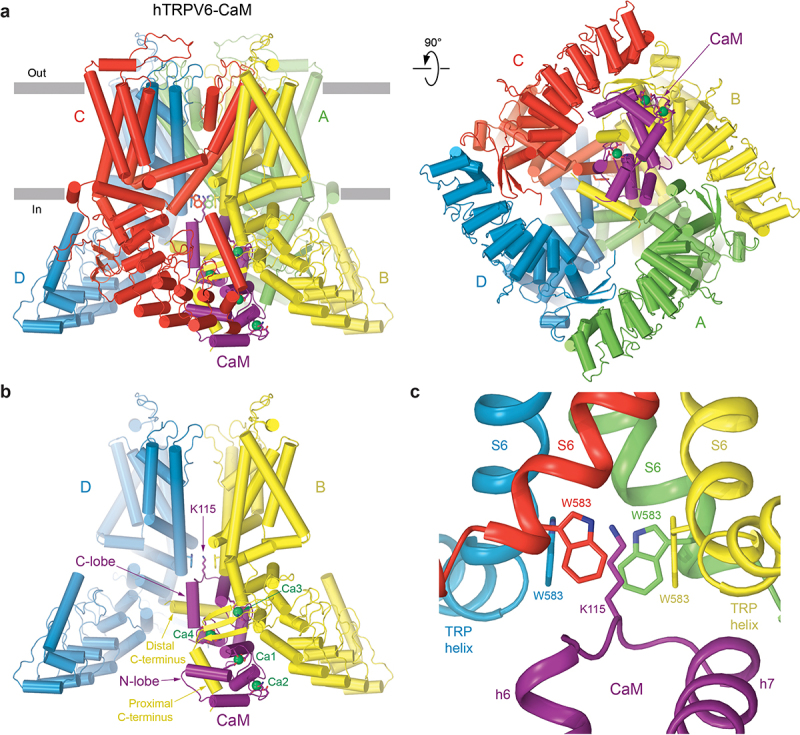
**Structure of inactivated TRPV6 in complex with calmodulin.** a, side (left) and bottom (right) views of hTRPV6-CaM complex (PDB ID: 6E2F), with hTRPV6 subunits (A-D) colored green, yellow, red, and blue and CaM colored purple. Calcium ions are shown as green spheres. Side chains of hTRPV6 residues W583, CaM residue K115 and those that coordinate calcium ions are shown as sticks. b, side view of hTRPV6-CaM, with only two of four subunits shown and the front and back subunits removed for clarity. c, expanded view of the intracellular pore entrance, with CaM residue K115 forming a unique cation–π interaction with the cubic cage of side chain indoles contributed by W583 from each TRPV6 subunit. Adapted from [14].

TRPV6 is also endogenously regulated by the calcium-binding protein calmodulin (CaM), which mediates calcium-induced inactivation of this channel [11,12,14,76,77] as well as its close relative TRPV5 [77–81]. CaM docks underneath the intracellular skirt of TRPV6 after its *N*- and C-lobes, each binding two Ca^2+^ ions, adapt a distinct head-to-tail arrangement [14] (Figure 3a, b). CaM inserts its lysine residue 115 into the intracellular pore entry site [69], where four highly conserved tryptophan W583 residues, one from each subunit, form a closely packed cubic structure with the distance of 4.2 Å between the planes of the oppositely located tryptophan indole rings (Figure 3c). This arrangement of tryptophan side chains facilitates a particularly strong cation-π interaction with the positively charged ε-amino group of lysine K115. The significance of W583 in CaM-dependent calcium-induced inactivation has been demonstrated in experiments with TRPV5, where mutations such as W583A resulted in cell death due to increased calcium influx [82]. Mutations of W583 to leucine or alanine significantly reduced CaM-mediated inactivation of TRPV5 and caused the channel to remain in the open state [80,82]. Strong cation-π interaction between W583 residues of TRPV6 and CaM residue K115 results in pore narrowing that brings isoleucines I575 close to each other to hydrophobically seal the channel.

### Magnesium

Strong inward rectification of TRPV6-mediated current [9,10] can be partly attributed to inhibition of the channel by intracellular Mg^2+^. However, little is known about the molecular mechanism of TRPV6-regulation by magnesium ions, including the lack of knowledge about putative Mg^2+^ binding sites. It is likely that magnesium ions regulate TRPV6 through the mechanism of ion channel block (by plugging the pore, like in NMDA receptor channels [83,84]) or allosteric inhibition via an unidentified intracellular binding site. Alternatively, Mg^2+^ may be binding to the negatively charged head group of PIP_2_ and sequestering the amount of free PIP_2_ available for TRPV6 activation, similar to what was proposed for KCNQ potassium channels [85].

## Synthetic inhibitors of TRPV6

Pharmacological search for TRPV6 inhibitors can, in principle, follow different pathways: (1) *de novo* design of new small molecules based on available molecular structures of TRPV6, (2) design of new molecules based on already existing TRPV6 inhibitors, through chemical modification aimed at improving their affinity and specificity, and (3) screening of available libraries of various compounds with the hope that some of these compounds will turn out to be potent and selective inhibitors of TRPV6. So far, the second and third pathways have been most productive and below we give examples of some of the discovered small molecules that inhibit TRPV6 according to various molecular mechanisms.

### Ion channel blockers

#### Inactivation-mimicking PCHPDs

**Figure 4.**
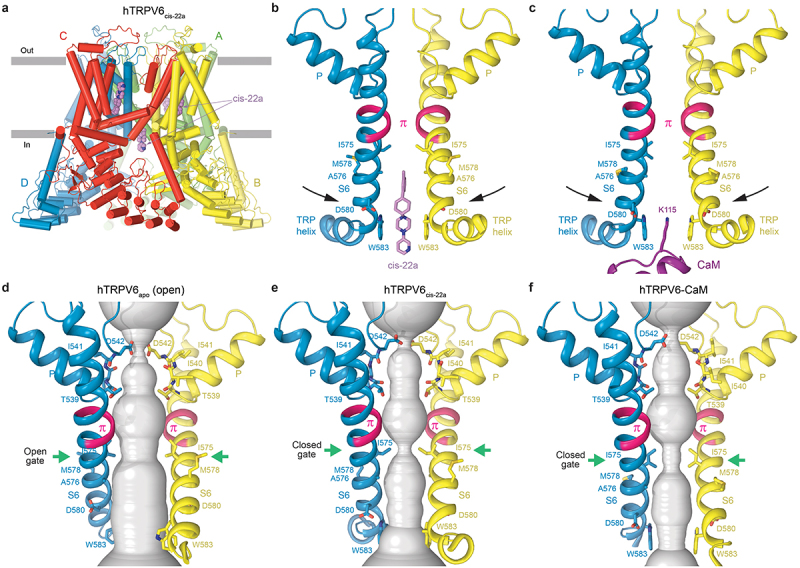
**Inactivation-mimicking block of TRPV6 by PCHPDs.** a, side view of hTRPV6_cis-22a_ structure, with hTRPV6 subunits (A-D) colored green, yellow, red, and blue and the PCHPD cis-22a molecules shown as space-filling models (violet). b,c, pore-forming domains in hTRPV6_cis-22a_ (b, PDB ID: 7K4B) and hTRPV6-CaM (c, PDB ID: 6E2F). Only two of four hTRPV6 subunits are shown, with the front and back subunits removed for clarity. The region undergoing α-to-π transition in the middle of S6 is colored pink. The molecule of cis-22a (b, violet), CaM residue K115 (c, purple) and TRPV6 residues around the gate are shown as stick models. d – f, hTRPV6 ion conduction pathway (gray) in the open-state structure hTRPV6_apo_ (d, PDB ID: 7K4A) and inactivated-state structures hTRPV6_cis-22a_ (e, PDB ID: 7K4B) and hTRPV6-CaM (f, PDB ID: 6E2F). The gate region is indicated by green arrows. Adapted from [64].

Aiming to design TRPV6 inhibitors targeting the vanilloid pocket, which was first structurally characterized in TRPV1 channels [86,87] and found to serve as an allosteric inhibition site for TRPV1 by analgesic molecules [88], a team of scientists developed a series of PCHPDs that turned out to be highly selective, nanomolar-affinity TRPV6 inhibitors [56–58]. X-ray and cryo-EM structures of TRPV6 in the presence of PCHPDs [64] indeed detected binding of these inhibitors to the vanilloid site [69,87], which is located in the TMD region facing the cytoplasmic leaflet of the membrane, in the crevice between S1–S4 and pore domains, contributed by S3, S4, S4–S5 linker, S5 and S6, and domains at the TMD-skirt interface, including LH2, S2-S3 loop and TRP helix (Figure 4a). At the vanilloid site, PCHPD replaces a cholesteryl hemisuccinate (CHS) lipid molecule, which is typically added during TRPV6 purification [62–66], and acts as a cholesterol molecule that occupies this site in natural conditions.

Despite the vanilloid site occupancy, the main PCHPD binding site in TRPV6 appeared to be the intracellular pore entry site [69] (Figure 4a, b), exactly where CaM binds (Figure 4c). The central role of the pore site was confirmed by mutagenesis of residues involved in PCHPD binding. Notably, the W583F mutation, which caused strong attenuation of CaM inactivation [80,82], also reduced PCHPD inhibition. D580K resulted in nearly complete elimination of PCHPD inhibition, indicating a critical role of this residue in the inhibitor binding. Sharing the same site makes PCHPDs and CaM competitors for binding to TRPV6. Assuming the dynamic nature of CaM-induced inactivation of TRPV6, highly potent PCHPDs should be able to outcompete CaM, which is continuously present inside the cell under physiological conditions.

The presence of PCHPD at the intracellular pore entrance causes similar conformational changes as those induced by CaM. Indeed, compared to the widely open pore of the apo-state TRPV6 (Figure 4d), the pores of PCHPD-bound (Figure 4e) and CaM-bound (Figure 4f) channels are much narrower, with the side chains of isoleucines I575 forming a hydrophobic seal. Interestingly, pore narrowing during CaM inactivation or inactivation-like block by PCHPDs is characterized by the preserved conformation of the S6 helix that, similar to the open state, contains a π-bulge in the middle. The non-conducting inactivated conformation of the ion channel pore is therefore very different from the closed state pore, characterized by the entirely α-helical S6 (see below) [63,65,89]. Thus, PCHPDs inhibit TRPV6 by mimicking the action of CaM and playing the role of gating modifying channel blockers [14]. In this case, strong cation–π interaction of the positively charged ε-amino group of CaM lysine K115 with the cubic cage of W583 indole rings during inactivation (Figure 3c) is replaced with aromatic interactions between the W583 cage and the ring system of PCHPDs as well as electrostatic interactions between the positively charged tertiary amine of PCHPD and negatively charged side chains of aspartates D580 (Figure 4b, c). The unique inactivation-mimicking mechanism of highly potent and selective TRPV6 inhibition by PCHPDs highlights a promising direction for the design of future-generation biomimetic drugs.

#### Ruthenium red

The inorganic dye ammoniated ruthenium oxychloride, more commonly known as ruthenium red (RR), is used in many biological applications [90–92]. RR inhibits a broad spectrum of ion channels, including TRP, calcium homeostasis modulator (CALHM), two-pore domain potassium (K2P), and Piezo channels as well as ryanodine receptors and mitochondrial calcium uniporters [93,94].

**Figure 5.**
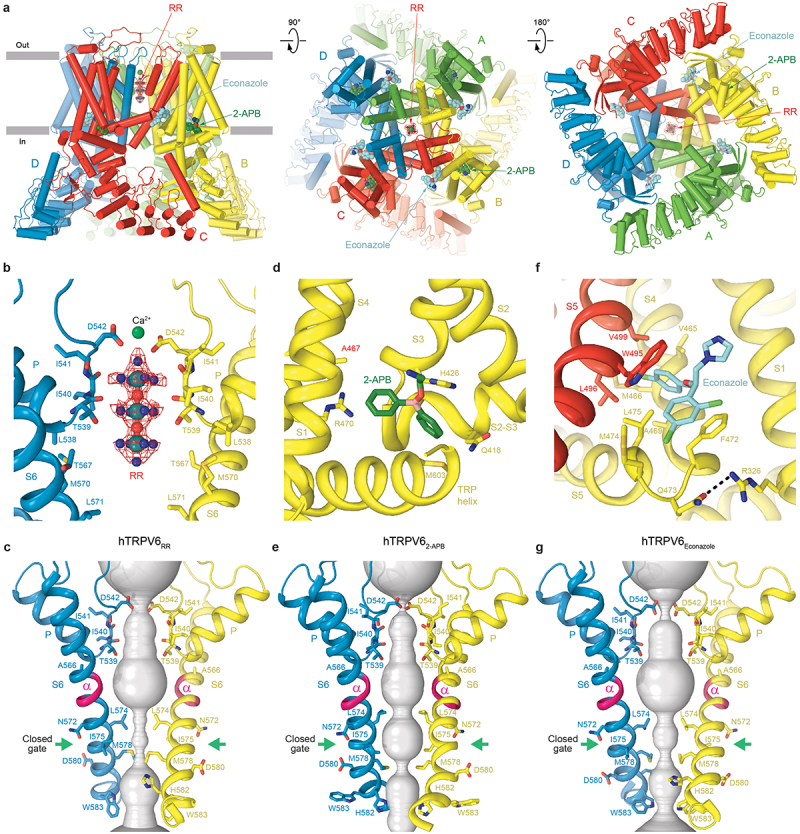
**Structures of TRPV6 in complex with synthetic inhibitors RR, 2-APB and econazole.** a, side (left), top (middle), and bottom (right) views of hTRPV6_RR_ (PDB ID: 7S8B), with hTRPV6 subunits (A-D) colored green, yellow, red, and blue. The RR molecule is shown as a ball-and-stick model, with the corresponding cryo-EM density shown as red mesh and Ca^2+^ ion as a green sphere. Molecules of 2-APB (dark green) from hTRPV6_2-APB_ (PDB ID: 6D7T) and econazole (cyan) from hTRPV6_Eco_ (PDB ID: 7S8C) are shown as space-filling models. b,d,f, expanded views of the RR (b), 2-APB (d) and econazole (f) binding sites. RR molecule is shown the same way as in a. Molecules of 2-APB (dark green) and econazole (cyan) as well as residues contributing to inhibitor binding are shown as stick models. c,e,g, ion conduction pathway (gray) in hTRPV6 bound to RR (c), 2-APB (e) and econazole (g), with residues lining the selectivity filter and around the gate shown as stick models. Only two of four subunits are shown, with the front and back subunits removed for clarity. The region undergoing α-to-π transition in the middle of S6 is colored pink. The gate region is indicated by green arrows. Adapted from [65,89].

Using cryo-EM, calcium imaging, and mutagenesis, it was shown that RR acts as a TRPV6 ion channel blocker [65]. RR binds to the selectivity filter site [69] that also represents a binding site for permeant ions (Figure 5a, b). A high, near-atomic resolution of the hTRPV6_RR_ structure allowed an accurate description of the RR binding position, which on the extracellular side is flanked by four aspartates D542 surrounding a spherical density in the middle, likely representing a calcium ion (Figure 5b). The inhibitor occupies the entire selectivity filter of TRPV6, being coordinated by the carboxyl groups of D542, backbone carbonyl oxygens of I541, I540, and T539, and hydroxyl group of T539, and spans the upper half of the pore’s central cavity. The surface of the selectivity filter has a strong negative charge, providing a favorable environment for RR, which carries a total of +6 positive charge.

The pore of hTRPV6_RR_ is hydrophobically sealed by side chains of L574 and M578 in the intracellular gate region (Figure 5c). In contrast to the open and inactivated states, which have a π-bulge in the middle of S6 (Figure 4d-f), S6 in hTRPV6_RR_ is entirely α-helical (Figure 5c), typical for the closed conformation of TRPV6. The transition from the open to closed state is accompanied by a ~ 100-degree rotation of the S6 intracellular portion [63]. Accordingly, the side chains of isoleucines I575, which determine the gate region in the open and inactivated states (Figure 4d-f), turn away and become substituted with side chains of L574 and M578 that now determine the gate region in the closed state (Figure 5c).

The conformational change in S6 starts below the gating hinge alanine A566 and does not disrupt the upper pore of TRPV6, thereby leaving the selectivity filter essentially intact. How does RR, which binds in the selectivity filter without altering its conformation, cause structural changes in the intracellular gate region of the pore? It was hypothesized that the positively charged RR creates an electric field within the central cavity of the pore, which interacts with the electric dipole of the S6 helix [65,89]. This field causes a repulsion of the lower portion of S6 away from RR and its rotation. As a result of this rotation, S6 becomes completely α-helical, with the side chains of L574 and M578 sealing the channel pore.

### Allosteric modulators

#### 2-APB

2-aminoethoxydiphenyl borate (2-APB), a small molecule that can penetrate cell membranes, is one of a few compounds known to act as inhibitors of TRPV6. In human cancer cell lines of cutaneous squamous cell carcinoma, 2-APB demonstrated the ability to reduce tumor growth and invasiveness in vitro [95]. Initially identified as an inhibitor of inositol 1,4,5-trisphosphate (InsP3) receptor-induced Ca^2+^ release [96], 2-APB was subsequently found to inhibit various TRP channels [89,97–99], including TRPV6 [89]. The structure of TRPV6 in complex with 2-APB [89] revealed the inhibitor binding to the S1–S4 base site [69] (Figure 5a, d). In the absence of 2-APB, this site is occupied by a CHS lipid molecule that is used during TRPV6 purification, which likely substitutes a molecule of cholesterol that resides in this site in natural conditions [62–66].

2-APB binding not only causes the dissociation of the resident lipid, but it also leads to the formation of a hydrophobic cluster of residues contributed by S3, S4, and the S4-S5 linker [89]. The cluster formation is accompanied by rearrangement of transmembrane helices that results in the disruption of hydrogen bonds between Q473 in the S4–S5 linker and R589 in the TRP helix, as well as between D489 in the S5 helix and T581 in the S6 helix. These hydrogen bonds stabilize the open state by energetically compensating the unfavorable α-to-π transition in the middle of S6. Consequently, the hydrogen bonds removal makes S6 α-helical, the ~100-degree rotated intracellular portions of S6 bring the side chains of L574 and M578 to the center of the ion channel pore, which in turn create a hydrophobic seal and finalize the transition of the channel to the closed non-conducting state (Figure 5e), similar to the state of the hTRPV6_RR_ structure (Figure 5c).

#### Econazole

Econazole is an FDA-approved anti-fungal agent [100–102], which has been proposed for repurposing as a TRPV6 antagonist [65,103,104]. In fact, repurposing is an efficient way to increase the drug’s portfolio, given that these compounds have already proven themselves to be safe in humans [105]. This is especially important in the drug development market, where the success rate for launching a new drug is diminishingly small due to three key factors: (1) unpredictability of which compounds in a pool of candidates in both pre-clinical screenings and in clinical development will eventually reach authorization for market launch, (2) information and resource asymmetries between the seller of a clinical drug candidate in development (smaller and typically less resourceful biotech companies who have better knowledge on the true value of that drug candidate) and the buyer (a larger biotech or pharmaceutical company that normally lacks full insight into the likelihood of the drug reaching the market), and (3) regulatory (i.e. FDA approval guidelines) and financial (prioritization of certain lead compounds in a pricey clinical drug development process) bottlenecks [106].

Cryo-EM studies of TRPV6 in complex with econazole [65] revealed its binding to the shallow S4-S5 site [69] at the junction between S5 of one subunit and S4 of the adjacent subunit (Figure 5a, f). Within this site, econazole is positioned between W495 of S5 and F472 of S4, surrounded by hydrophobic amino acids including L496 and V499 of S5, as well as M466, A469, M474, and L475 of S4. Not surprisingly, the hydrophobic nature of this binding site makes it a favorable environment for a lipid, which binds to this site in the absence of econazole. Validation of this novel econazole binding site using mutagenesis and calcium uptake measurements identified W495A and F472A mutations that had a significant impact on econazole inhibition and confirmed that the binding site at the S4–S5 interface is the primary site of econazole inhibition.

The smaller size of econazole in comparison to the lipid creates a vacant space that allows the side chains of Q473 and M474 to move closer to F472 and W495, respectively, while moving away from R589. The separation of Q473 and R589 causes the disruption of the open state, stabilizing a hydrogen bond between them. The loss of this hydrogen bond, which usually compensates for the energetically unfavorable α-to-π transition in S6, leads to the reversal of this transition. As a result, the lower portions of the S6 helices undergo a ~ 100° rotation, resulting in the separation of D489 and T581 and the consequent loss of the open state-stabilizing hydrogen bond between them, while the side chains of L574 and M578 become exposed toward the center of the pore, hydrophobically sealing the pore and preventing the passage of ions. This transformation therefore converts the channel into the closed, non-conducting state (Figure 5g).

## Natural inhibitors of TRPV6

Synthetic inhibitors of TRPV6 have been making a rather slow progress toward clinical trials [5,15,16,64,65,107]. In the meanwhile, the scientific exploration and pharmaceutical exploitation of natural inhibitors for TRPV6 have been somewhat overlooked. Nevertheless, natural compounds have a great potential as their pharmacokinetics has already been optimized by nature in the course of evolution [108]. The molecular mechanism of natural compounds inhibiting TRP channels, including those that have been used in traditional medicine, has been successfully studied by cryo-EM [66,109,110], demonstrating the power of this technique for structural pharmacology.

### Phytocannabinoid tetrahydrocannabivarin

For thousands of years, humans have utilized *Cannabis sativa* preparations for medicinal purposes [111]. However, due to the stigma surrounding cannabis and legal restrictions aimed at preventing drug abuse, utilization of the therapeutic potential of various phytocannabinoids has been significantly delayed. In recent times, there has been a shift in perception and an easing of legal barriers, allowing for a reevaluation of cannabis, its natural products and synthetic analogs. Despite many of these substances being now considered for therapeutic applications, the mechanisms of their action remain poorly understood. Numerous human diseases, including cancer, anorexia, emesis, pain, inflammation, multiple sclerosis, neurodegenerative disorders (such as Parkinson’s disease, Huntington’s disease, Tourette’s syndrome, and Alzheimer’s disease), epilepsy, glaucoma, osteoporosis, schizophrenia, cardiovascular disorders, obesity, and metabolic syndrome-related disorders, are either being treated or have a potential to be treated with cannabinoid-based bioactive compounds [112]. Notably, cannabinoids and their analogs have been shown to target a variety of TRP channels, including TRPV1–4, TRPA1, and TRPM8 channels, for therapeutic purposes [113].

**Figure 6.**
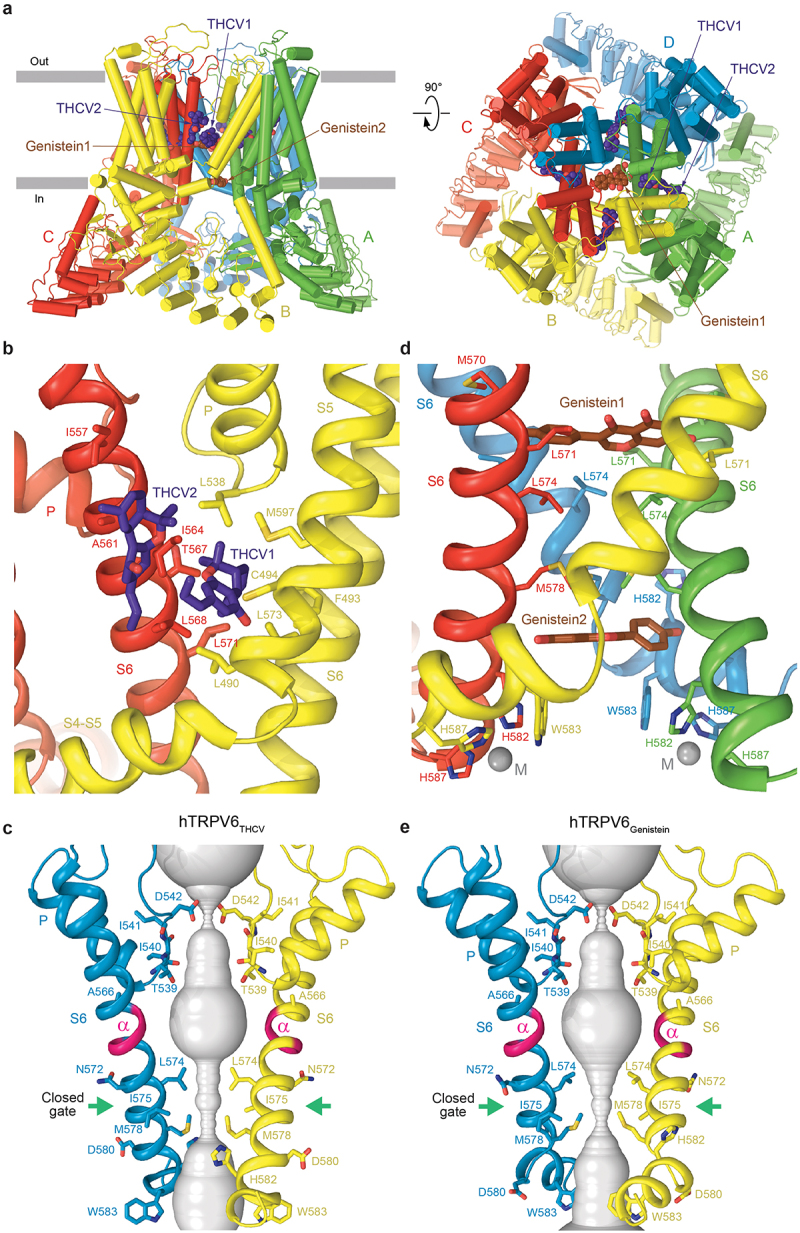
**Structures of TRPV6 in complex with natural inhibitors THCV and genistein.** a, side (left) and top (right) views of hTRPV6_Gen_ (PDB ID: 8FOA), with hTRPV6 subunits (A-D) colored green, yellow, red, and blue. Molecules of genistein (brown) and THCV (purple) from hTRPV6_THCV_ (PDB ID: 8SP8) are shown as space-filling models. b,d, expanded views of the THCV (b) and genistein (d) binding sites. THCV (purple) and genistein (brown) molecules are shown as stick models. c,e, ion conduction pathway (gray) in hTRPV6 bound to THCV (c) and genistein (e), with residues lining the selectivity filter and around the gate shown as stick models. Only two of four subunits are shown, with the front and back subunits removed for clarity. The region undergoing α-to-π transition in the middle of S6 is colored pink. The gate region is indicated by green arrows. Adapted from [66,110].

A recent structural study of human TRPV6 inhibition by a phytocannabinoid tetrahydrocannabivarin (THCV), a naturally occurring non-psychoactive analog of tetrahydrocannabinol [110], revealed binding of this inhibitor to the deep and shallow portal sites [69] that connect the channel pore to the surrounding membrane environment (Figure 6a, b). For THCV to bind to site 1, the surrounding side chains need to move to create a space between S5 and S6 of the neighboring subunits. These adjustments mainly involve F493 and M497 on S5, which change their side-chain conformations. In addition, the entire section of the S4-S5 linker, located before F493, undergoes translation and rotation to accommodate THCV binding.

Compared to the open or inactivated states, the N-terminal part of S6 and the pore loop in hTRPV6_THCV_ remain unchanged, while the C-terminal part of S6 undergoes a ~ 100° rotation, with the side chains of T567, L568, and L571 making contacts with THCV. The rotation also reverses the α-to-π transition in S6 that happens upon channel opening and makes S6 entirely α-helical. Concomitantly, S6 becomes two helical turns shorter, while the TRP helix two helical turns longer. Additionally, this rotation repositions M578 so that its side chain points toward the channel center and hydrophobically seals the pore, preventing water and ion conductance and completing the transition from the open to the closed antagonist-bound state (Figure 6c).

The THCV binding sites are different from binding sites of all other known TRPV6 ligands that have been characterized structurally. Among the 14 identified ligand-binding sites in TRPV channels [107], this specific deep portal site has been previously found to bind the agonist cannabidiol (CBD) in TRPV2 and the local anesthetic dyclonine in TRPV3 [114–116]. Dyclonine, however, penetrates further into the portal site and extends into the channel pore, presumably creating a direct barrier that hinders the flow of ions and water. In contrast, CBD does not extend into the TRPV2 pore but, compared to THCV inhibition of TRPV6, produces an opposite allosteric action by causing channel opening. Interestingly, in contrast to THCV, CBD and other cannabinoids have been shown to be ineffective as antagonists of TRPV5 and TRPV6 in previous studies, at least at the doses tested [117]. Compared to dyclonine in TRPV3, THCV exhibits TRPV6 according to an allosteric mechanism unique among TRPV channels. According to this mechanism, THCV stabilizes the closed state of TRPV6 by acting as a molecular cog inserted into the cogwheel mechanism of the channel gating.

### Isoflavone genistein

The natural isoflavone and phytoestrogen genistein (4’,5,7-trihydroxyisoflavone) extracted from *Styphnolobium japonicum* has been shown to act as a strong TRPV6 inhibitor [104]. Genistein is a precursor in the biosynthesis of antimicrobial compounds phytoalexins and phytoanticipins in legumes and, as a predominant isoflavone in nutritional soy, can be a major component of an individual’s diet [118–121]. Indeed, dietary genistein shows a range of potential health benefits, including inhibition of cell invasion and metastasis in various forms of cancer [118,119,122–130]. Beyond its potential for the treatment of prostate, colon, kidney, pancreatic, ovarian, breast and lung cancers [125,131–154], putative therapeutic value of genistein extends to treatment of cardiovascular diseases [155–157], post-menopausal [158,159] and gastrointestinal [160] ailments and bone loss [161–164]. Genistein has already been investigated in 75 clinical trials (clinicaltrials.gov), which demonstrated its antimetastatic efficacy [165] and positive effects in treatment of metabolic syndrome [166].

A recent study using cryo-EM combined with calcium imaging, electrophysiology, mutagenesis, and MD simulations [66], showed that genistein binds in the intracellular half of the hTRPV6 pore, including the intracellular pore entry site [69] where CaM [14] and PCHPDs [64] bind, and acts as an ion channel blocker and gating modifier (Figure 6a, d). When genistein molecules bind to the open TRPV6 channel, they do so perpendicularly to the central pore axis, causing an asymmetrical pulling of one diagonal pair of subunits toward the channel center. This motion disrupts the interactions between residues D489 in S5 and T581 in S6, as well as Q473 in the S4-S5 linker and R589 in the TRP helix. These interactions are crucial for stabilizing the energetically unfavorable α-to-π transition in S6 and maintaining the open-pore conformation. As a result of genistein binding, S6 undergoes a reverse π-to-α transition, accompanied by a ~ 100-degree rotation of its intracellular portion (Figure 6e).

While interactions with genistein molecules do not alter S6 helices in subunits A and C, the TRP helices become shorter due to unwinding of their N-terminal portions. To match these structural changes, the S6-TRP helix region in subunits B and D incorporates an additional short helix between S6 and the TRP helix. Concomitantly, the S4-S5 region in subunits A and C changes the angle between the S4-S5 helical linker and S5 and includes an unfolded segment following S4, while in subunits B and D, it incorporates an unfolded segment between the S4-S5 helical linker and S5. Interestingly, all these conformational changes are localized to the channel’s intracellular core and do not extend beyond the S4–S5 and S6-TRP helix regions. The rest of the TRPV6 molecule remains essentially unchanged. The precise contribution of two genistein binding sites (sites 1 and 2) to the mechanism of TRPV6 inhibition remains unclear. Given the more stable behavior in MD simulations, site 1 was proposed to be the main site [66]. Correspondingly, mobility of genistein at site 2 and a relatively weak contribution of metal coordination in the vicinity of site 2 suggest that this site plays a secondary role in TRPV6 inhibition.

## Discussion

TRPV6 plays a crucial role as a regulator of calcium balance in mammals. When the normal TRPV6 function is disturbed, it causes changes in calcium homeostasis, leading to various human diseases, including different forms of cancers. Consequently, there is a pressing need for inhibitors of this oncochannel. In this review, we presented an overview of recent advances in the molecular pharmacology of TRPV6. These advances have shed light on the molecular mechanisms behind TRPV6 inhibition by a range of small-molecule compounds, including approved drugs and natural agents derived from plants.

**Figure 7.**
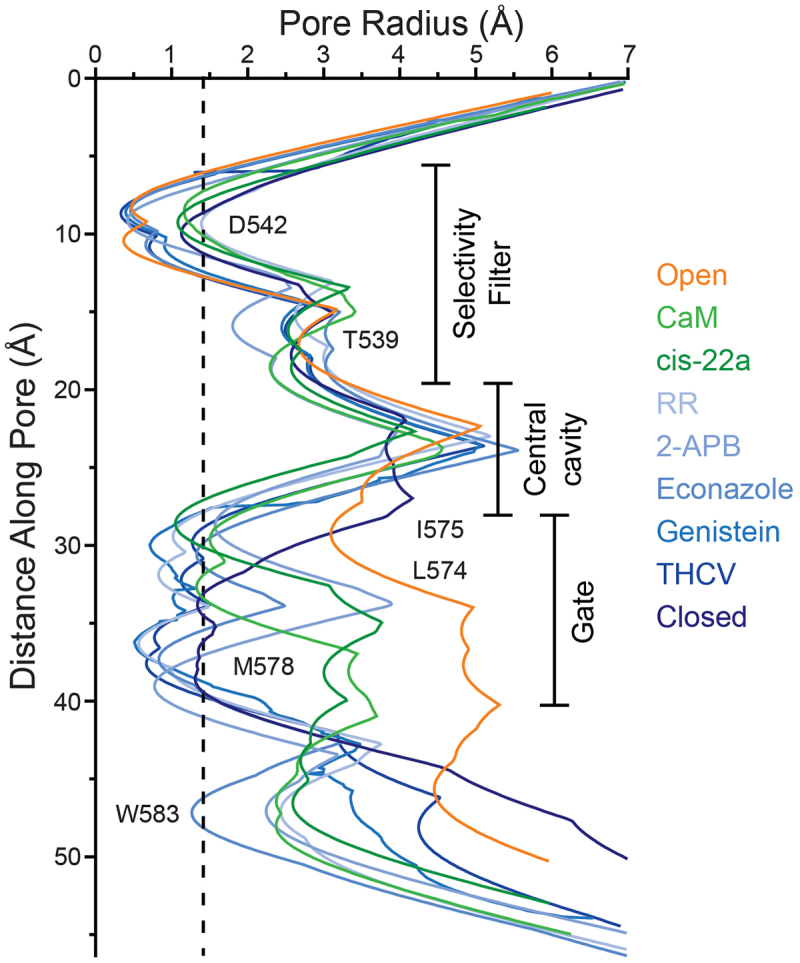
**Pore radius in the closed, open and inactivated states**. Pore radius calculated using HOLE [167] for hTRPV6 in the open (orange; PDB ID: 7K4A), inactivated by CaM (light green, PDB ID: 6E2F) and cis-22a (dark green, PDB ID: 7K4B), and closed (shades of blue, from light to dark, for RR, PDB ID: 7S8B; 2-APB, PDB ID: 6D7T; econazole, PDB ID: 7S8C; genistein, PDB ID: 8FOA; THCV, PDB ID: 8SP8; R470E mutant, PDB ID: 6BOA) states. Adapted from [14,63–66,89,110].

Based on structural studies, there are two major mechanisms of TRPV6 inhibition: direct block by occluding the ion channel pore (PCHPDs, RR, genistein, Ga^3+^, calmodulin) and allosteric inhibition accompanied by the displacement of lipids (2-APB, econazole). THCV likely also belongs to the second category, as it occupies the space that can house the tail of a putative lipid. Except for PCHPDs and CaM, which transition the channel into the inactivated state, all other structurally investigated inhibitors lock the channel in the closed state, characterized by the α-helical conformation of S6. Hence, despite differences in binding site locations, interactions with lipids and local conformational rearrangements, the pore of TRPV6 appears to favor three major architectures, open, closed, and inactivated, that are characterized by three distinct types of pore radius profiles (Figure 7).

Most if not all inhibitor binding sites and inhibitory mechanisms are not unique to TRPV6 and can be found in other TRP channels or other channel families [69]. Further research and solving more high-resolution structures will likely identify new sites for drug targeting on the surface of TRPV6. New advances in structural biology such as *in situ* cryo-electron tomography along with native protein preparations are expected to broaden our understanding of TRPV6 regulation beyond the single-protein level. The combination of new and traditional approaches will provide better understanding of the mechanisms and energetics of TRPV6 inhibition.

## Data Availability

The authors confirm that the data supporting the findings of this study are available within the article.
